# Imaging Accuracy in Preoperative Staging of T3-T4 Laryngeal Cancers

**DOI:** 10.3390/cancers12051074

**Published:** 2020-04-26

**Authors:** Marco Benazzo, Fabio Sovardi, Lorenzo Preda, Simone Mauramati, Sergio Carnevale, Giulia Bertino, Francesca Berton, Matteo Meroni, Irene Herman, Giuseppe Trisolini, Patrizia Morbini

**Affiliations:** 1 Department of Otorhinolaryngology, University of Pavia, IRCCS Policlinico San Matteo Foundation, 27100 Pavia, Italy; marco.benazzo@unipv.it (M.B.); simone.mauramati@gmail.com (S.M.); g.bertino@smatteo.pv.it (G.B.); meroni369@gmail.com (M.M.); herman.ire@gmail.com (I.H.); giuseppe.trisolini01@universitadipavia.it (G.T.); 2 Radiology Department, University of Pavia, IRCCS Policlinico San Matteo Foundation, 27100 Pavia, Italy; l.preda@smatteo.pv.it (L.P.); f.berton@smatteo.pv.it (F.B.); 3 Section of Anatomic Pathology, Cerba Healthcare Italia, 20139 Milan, Italy; rem686@hotmail.it; 4 Unit of Pathology, Department of Molecular Medicine, University of Pavia, IRCCS Policlinico San Matteo Foundation, 27100 Pavia, Italy; patrizia.morbini@unipv.it

**Keywords:** laryngeal cancers, TNM staging, standard CT, glottic map, imaging accuracy

## Abstract

Background: Preoperative imaging impacts treatment planning and prognosis in laryngeal cancers. We investigated the accuracy of standard computed tomography (CT) in evaluating tumor invasions at critical glottic areas. Methods: CT scans of glottic cancers treated by partial or total laryngectomy between Jan 2015 and Aug 2019 were reviewed to assess levels of tumor invasion at critical glottic subsites. CT accuracy in the identification of tumor extensions was determined against the gold standard of histopathological analysis of surgical samples. Results: This study included 64 patients. In the anterior commissure, CT showed high rates of false positives at all levels (sensitivity 56.2–70%, specificity 87.8–92.3%); in the anterior vocal fold, it overestimated the deep invasion (19.5% specificity, 90.3% sensitivity), while it underestimated the extralaryngeal spread (63.6% sensitivity, 98.1% specificity). In the posterior paraglottic space (pPGS), false negative results were more frequent for superficial extensions (25% sensitivity, 95.8% specificity) and deep invasions (58.8% sensitivity, 82.3% specificity). Shorter disease-specific and disease-free survivals were associated with pStage IV (*p*: 0.045 and 0.008) and with the pathological involvement of pPGS (*p*: 0.045 and 0.015). Conclusions: Negative prognostic correlation of pPGS involvement was confirmed on histopathological data. CT staging did not provide a satisfactory prognostic stratification and should be complemented with magnetic resonance imaging.

## 1. Introduction

Advanced laryngeal cancers (T3–T4a) is a broad category comprising lesions that have heterogeneous patterns of spread and biologic behaviors despite their relative histological homogeneity (squamous cell carcinoma, SCC). This heterogeneity may be attributed to the different characteristics of laryngeal anatomical subsites. Fat tissue is a weak barrier against the tumoral spread, while other structures, such as cartilages and ligaments, can play an important role in limiting neoplastic progression. Vessels and lymphatics, more abundant in the supraglottic region than in the glottis and subglottis, also have great roles in cancer progression.

Focusing on glottic cancers, the main pattern of tumor spread is by local extension; in fact, since the true vocal folds have a very sparse lymphatics drainage, these cancers tend not to spread via lymphatics until the tumor has extended beyond the vocal fold. Different scenarios can be recognized based on the glottic subsite and compartments involved. Cancers originating from the anterior commissure (AC) may spread anteriorly through the Broyle’s ligament and the thyroid cartilage, reaching the infrahyoid muscles, cranially to the pre-epiglottic space (PES) and to the thyro-hyoid membrane, caudally to the cricothyroid membrane and postero-laterally involving the vocal cord. Tumors that originate from the vocal cord may extend laterally through the thyro-arytenoid muscle, the paraglottic space (PGS) and the thyroid cartilage; moreover, they can extend superficially, along a cranio-caudal and antero-posterior axis.

In the American Joint Committee on Cancer (AJCC) TNM staging system, each T subcategory is based on specific tumor features, principally location and extension [1]. However, important differences may exist among tumors classified under the same T category. The T3 category of laryngeal tumors comprises all endo-laryngeal cancers with vocal cord fixations. Bulky tumor sizes or the involvement of different laryngeal structures may lead to this condition, with different clinical implications. Despite this evidence, no further subclassification has been validated so far. Recently, some authors highlighted a significant negative correlation between the involvement of the posterior (p)PGS (defined according to a plane passing through the arytenoid vocal process, perpendicular to the ipsilateral thyroid lamina) and prognosis [2,3]. This consideration has led to the suggestion that distinguishing the T3 category into two subcategories (with or without pPGS involvement) could improve the TNM predictive value. 

Moreover, the increasing number of surgical options has raised the attention to laryngeal subsites and their implications in tumoral pathways of spread. This has led to the introduction of endo-laryngeal compartment classifications to guide the treatment and better estimate patient prognosis [4,5]. Conservative surgical techniques, such as transoral laser microsurgery (TLM) [6,7,8] and open partial horizontal laryngectomies (OPHL) [5], can be applied for glottic cT3 tumors. The radiological evidence of pPGS involvement has been identified as the critical parameter in surgical decision algorithms in glottic T3 tumors [9]. Even if recent studies have shown the importance of magnetic resonance imaging (MRI) in glottic tumor preoperative staging and assessment [9,10], contrast-enhanced computed tomography (CT) remains the most commonly used imaging method in clinical settings [9,11,12,13].

The aim of this study is to assess the accuracy of standard CT imaging in the evaluation of tumor invasions of critical areas of the glottic region, which can change the therapeutic decision and are being considered for an upcoming revision of the TNM staging of laryngeal SCC. 

## 2. Results

The study included 64 patients (M:F ratio 5:1). The average age at surgery was 65.5 ± 9.2 years. Twenty-five patients (39.1%) had been submitted to total laryngectomy (TL), and 39 (60.9%) received OPHL. Eight of the OPHL patients (20.5%) underwent a subsequent TL, three (7.7%) for positive resection margins, three (7.7%) for laryngeal dysfunction and two (5.1%) for disease relapse. Among the TL patients, four (6.25%) were treated for disease recurrence after previous radiotherapy. Fifteen patients (23.4%) underwent post-surgical therapy: 2 (3.1%) chemotherapy, 10 (15.6%) radiotherapy and 3 (4.7%) chemoradiotherapy. 

Mean follow-up was 38.1 ± 33.8 months. Four patients (6.3%) were lost to follow-up. Twelve patients died (18.8%), six (9.4%) for disease recurrence and six (9.4%) for other causes; 54 patients showed no evidence of disease at their last follow-up (84.4%). 

**Figure 1:**
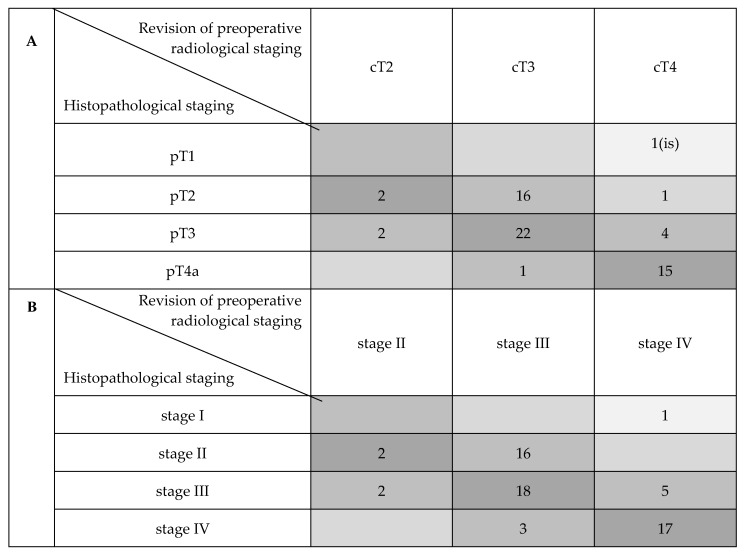
Compared distribution of cT with pT and cTNM with pTNM stages (AJCC 2017). (**A**) Comparison of cT and pT distribution. (**B**) Comparison of cTNM and pTNM stage distribution.

Table 1 shows the distribution of reviewed CT and histopathologic tumor staging. On the basis of imaging and a histopathologic sample review, the involvement of the anterior commissure (AC), anterior vocal fold (AVF) and posterior vocal fold (PVF) was assessed and compared with the help of radiological glottic maps (rMAPs) and histopathological glottic maps (pMAPs). Figure 1 summarizes the concordance between tumor staging as obtained with preoperative CT scanning and after a histopathological examination of the surgical specimens, highlighting the numbers of over-and under-staging.

In three of six radiologically over-staged cases (50%), a history of previous radiotherapy was described; the two radiologically down-staged cases were the result of false negative neck lymph nodes on CT scans. 

The overall diagnostic accuracy of the CT scan was 64.06% (95% confidence interval (CI) 51.1–75.68%) in the identification of T3 tumors, 89.06% (95% CI 78.75–95.49%) of T4a tumors, 59.38% (95 CI 46.38–71.49%) of tumor stage III and 85.94% (95% CI 74.98–93.36%) of tumor stage IV. 

**Figure 2:**
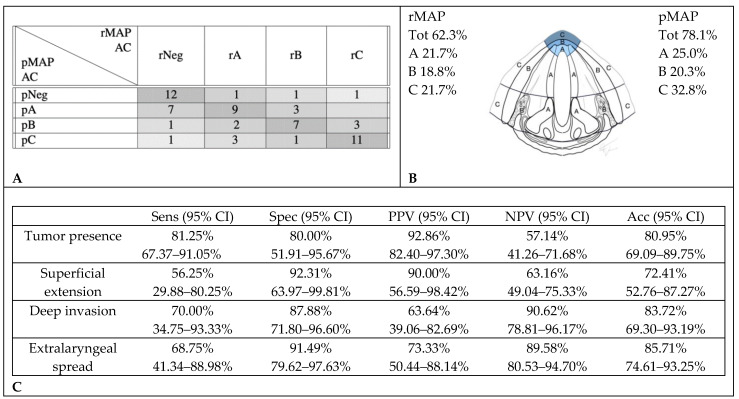
Accuracy in the radiological assessment of anterior commissure (AC) involvement. (**A**) Graphical representation of concordance between CT and histopathology in anterior commissure assessment. (**B**) Percentage of cancer involvement of anterior commissure in rMAP and pMAP. (**C**) Sensitivity (Sens), specificity (Spec), positive predictive value (PPV), negative predictive value (NPV) and accuracy (Acc) of CT in anterior commissure. CI: confidence interval, rMAP: radiological glottic map and pMAP: histopathological glottic map.

When analyzing the diagnostic accuracy for specific subsites, rMAP and pMAP comparison showed that CT scans correctly identified AC tumor involvement in 39 cases (61%) and over-staged tumor extension in nine cases (14.1%): in three cases, no tumor was found at the histopathological examination despite imaging suggestive of, respectively, a superficial, a deep endo-laryngeal and an extralaryngeal extension; in three cases, CT described deep invasion where only a superficial tumor was observed at histopathology, and in the last three, extralaryngeal extensions were reported where histological examination did not identify infiltration beyond the cartilage. Fifteen cases (23.4%) were under-staged: of nine patients negative on CT, seven were found to have histological superficial tumors, one deep infiltration and one extralaryngeal extension; among five cases with superficial tumors at CT, two were found to have histological deep infiltrations and three extralaryngeal extensions. Finally, in one case with deep endo-laryngeal infiltration on CT, the histological examination reported extralaryngeal extension (Figure 2). 

**Figure 3:**
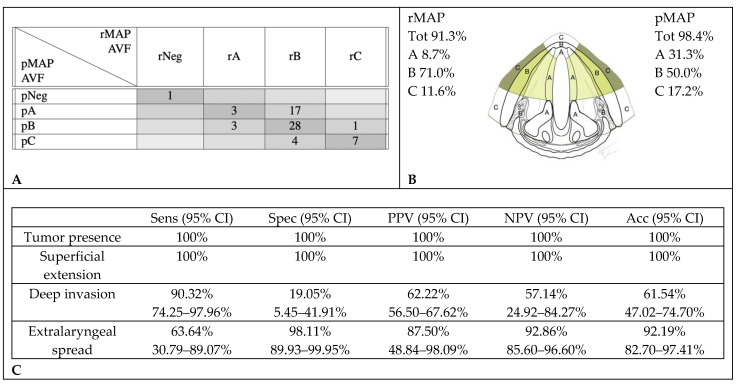
Accuracy in radiological assessment of anterior vocal fold (AVF) involvement. (**A**) Graphical representation of concordance between CT and histopathology in anterior vocal fold assessment. (**B**) Percentage of cancer involvement of anterior vocal fold in rMAP and pMAP. (**C**) Sensitivity (Sens), specificity (Spec), positive predictive value (PPV), negative predictive value (NPV) and accuracy (Acc) of CT in anterior vocal fold. CI: confidence interval, rMAP: radiological glottic map and pMAP: histopathological glottic map.

rMAP and pMAP agreed in the evaluation of the AVF and anterior (a) PGS involvement in 39 cases (61%). CT scans over-staged tumor extensions in 18 cases (28.1%): in 17 cases, it described deep invasions where only superficial tumors were observed at histopathology, and in the other one, it found an extralaryngeal extension where the histological examination did not identify infiltration beyond the cartilage. Seven cases (10.9%) were under-staged: three cases with superficial extensions on CT were found to have histological deep infiltrations, and four cases with deep infiltrations on CT were found to have histological extralaryngeal extensions (Figure 3). 

**Figure 4:**
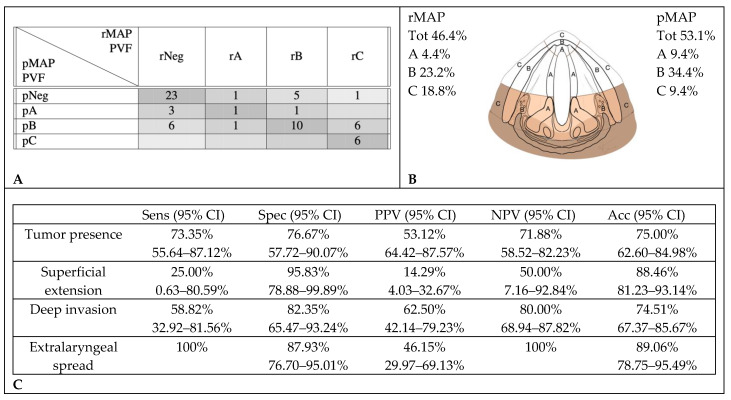
Accuracy in radiological assessment of posterior vocal fold (PVF) involvement. (**A**) Graphical representation of concordance between CT and histopathology in posterior vocal fold assessment. (**B**) Percentage of cancer involvement of posterior vocal fold in rMAP and pMAP. (**C**) Sensitivity (Sens), specificity (Spec), positive predictive value (PPV), negative predictive value (NPV) and accuracy (Acc) of CT in posterior vocal fold. CI: confidence interval, rMAP: radiological glottic map and pMAP: histopathological glottic map.

PVF and pPGS tumor involvement were correctly identified by CT scan reviews in 40 cases (62.5%). CT scans over-staged tumor extensions in 14 cases (21.9%): in seven, no tumor was found at the histopathological examination; in one, histopathology did not confirm a radiological deep invasion but only showed a superficial extension. In the last six cases, extralaryngeal invasions were down-staged to deep endolaryngeal extensions in pMAP. Ten cases (15.6%) were under-staged: of nine CT-negative cases, three were found to have histological superficial tumors and six, deep infiltrations; in the last one, with a superficial tumor on the CT, a deep infiltration was documented on histology (Figure 4).

**Figure 5:**
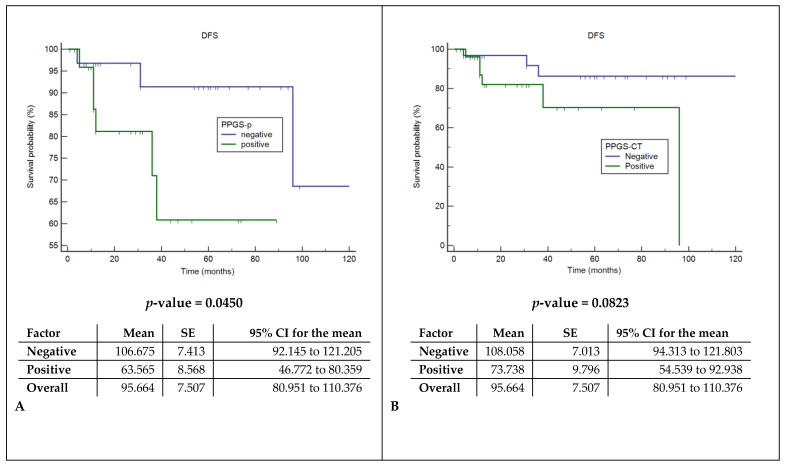

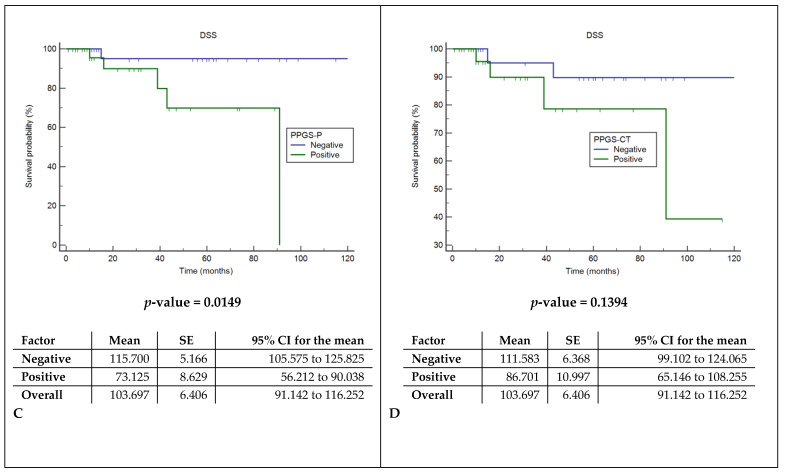
Disease-free survival (DFS) and disease-specific survival analysis (DSS). (**A**) Comparison of DFS between the histopathological assessment of spared or involved pPGS. (**B**) Comparison of DFS between the radiological assessment of spared or involved pPGS. (**C**) Comparison of DSS between the histopathological assessment of spared or involved pPGS. (**D**) Comparison of DSS between the radiological assessment of spared or involved pPGS. pPGS: posterior paraglottic space, SE: standard error and CI: confidence interval.

Cox regression models showed a significant correlation between the presence of pathologically documented tumor extensions in the pPGS and shorter DFS and DSS (*p*: 0.045 and 0.015), while CT invasions did not correlate with prognosis (Figure 5).

## 3. Discussion

Laryngeal squamous cell carcinoma is a very heterogenous disease, with remarkably different prognosis and therapeutic options depending on the tumor location and extension. For this reason, preoperative staging plays a key role in treatment planning, which is increasingly becoming more patient-tailored. Endoscopic evaluation under white light (WL) and narrow-band imaging (NBI) is considered the main diagnostic and staging tool for glottic SCC. According to the 8th edition of the AJCC tumor staging system, a vocal fold fixation represents the main parameter to classify cT3 tumors [1]. However, an endoscopic evaluation cannot assess the exact depth of invasion, nor the involvement of submucosal structures, such as paraglottic spaces, pre-epiglottic space or cartilage infiltration [14]. Currently, neck multidetector contrast-enhanced CT is the imaging technique recommended for laryngeal cancer staging. It is rapid, relatively cheap and it provides good resolution of the different anatomical structures. However, CT accuracy in correctly defining cancer extension is being debated. 

This study was focused on the diagnostic accuracy of CT imaging in assessing tumor involvement of specific laryngeal subsites. This topic has gained considerable importance not only for the role that a precise estimation of tumor extension has in treatment choice algorithms but also as a major prognostic factor. Recent studies have highlighted that pPGS and AC involvement seems to be associated with worse survival [2,3,4]. The main pitfalls of this study include the retrospective nature of the work and the limited number of enrolled patients, mainly due to the difficulty of obtaining high-quality preoperative CT scans. The main limits were the wide heterogeneity of CT scans available for review, which results from different acquisition protocols used in peripheral centers and from the evolution of imaging technologies over the last two decades.

Despite the above limitations, the comparison between radiological and histological evaluations of tumor extension parameters showed a frequent preoperative overestimation of the cancer spread. The main differences were noticed in the T3 category. This result could be attributed to the difficult discrimination between tumor and adjacent inflammation and edema, which may be mistaken for PGS involvement or early cartilage invasion. In the literature, CT overestimation in comparison with histopathological analysis is attributed to the lack of specific criteria for defining a cartilage invasion: Agada et al. reported over-staging in 45% of their study population, mainly due to the use of the arytenoid sclerosis alone as a radiological sign of cartilage invasion. Accuracy was enhanced to 71% when other parameters were also taken in consideration [15]. In our study, as in previously published papers [16], diagnostic accuracy was higher for the detection of T4 than of T3 tumors (89.1% vs. 64.1%). This suggests that extralaryngeal extension is more reliably recognized on CT than PGS or early cartilage involvement, which can be expressed mainly by cartilage sclerosis but also nonossification, lysis, erosions or obliteration of the medullary space. On the other hand, cartilage sclerosis is attributed to cancer osteoblastic activity related to the neoplastic infiltration, but it can be observed also when the tumor has not yet penetrated the perichondrium [16]. An interesting consideration is that the common occurrences of fibrosis and desmoplasia of the connective tissue surrounding irradiated tumors can be responsible for tumor over-staging and lower the diagnostic accuracy of recurrent disease after previous radiotherapy with respect to primary tumors (three out of five patients submitted to salvage laryngectomy in our series, one of whom, classified as cT4, was recognized as pTis). 

Tumor staging is aimed to stratify patients according to prognostic expectations. Therefore, new proposals of TNM classification should rely on the identification of improved isoprognostic categories. Succo et al. [2], in their proposal for a T3 subclassification of laryngeal cancer, suggested that tumor localization, within the same T3 category, is prognostically relevant: in particular, the invasion of the posterior laryngeal compartment correlated with a significantly worse prognosis. Del Bon et al. [3] also confirmed that the pT parameter has a lower prognostic accuracy than tumor localization. Piazza et al. [4] observed in TLM-treated T1-T3 glottic cancers that the AVF could be further subclassified into six distinct isoprognostic areas. The worst oncological outcomes results were found in pT2 or pT3 cancers with trans-AC extensions with or without involvement of the PES. Moreover, a significant decrease in the local control with TLM alone was observed at the passage from superficially confined neoplasms to cancers with superficial extensions of the tumors and, finally, to those with deep involvements of the laryngeal visceral spaces. These studies highlighted the prognostic role of tumor invasions in deep compartments of the larynx and, therefore, the importance of a precise preoperative assessment of cancer extensions for treatment planning; in particular, for the choice between conservative surgery (i.e., OPHL) and TL. 

To our knowledge, few studies compared laryngeal CT or MRI findings with the histological evaluation of surgical resection samples [15,16,17,18,19,20,21,22,23,24,25,26,27,28,29,30,31], and none of them focused on a systematic evaluation of glottic anatomical subsites. Instead, many CT-based studies considered only the detection of cartilage infiltration, while MRI-based ones mainly focused on PGS, PES and cartilage infiltration. From our data analysis, we observed that the overall accuracy of tumor detection by CT scan in the anterior commissure was 81.0%. In this area, as in the PVF, we observed a low sensitivity for superficially spreading tumors (56.3% and 25%). This result could be expected, since CT resolution does not easily recognize a flat superficial proliferation. In the AVF, instead, an alteration of the vocal fold profile due to superficial tumor growth was detected in all cases. It is obvious that CT limits in superficial evaluations are overcome by endoscopy (with NBI evaluations), which remains the essential exam for clinical staging. 

In the AVF, a tumor presence was always correctly identified. High sensitivity was observed for both superficial and deep intralaryngeal extensions (respectively, 100% and 90.3%), but false positives were very frequent in the evaluation of aPGS. The low specificity (19.1%) observed for deep endolaryngeal extensions may be considered an intrinsic limitation of CT scans. Even after contrast administration, the density of the tumor overlaps with that of adjacent soft tissues, making discrimination between normal and invaded structures troublesome. Moreover, the adipose tissue, which could facilitate the definition of deep tumor extension, is usually under-represented in aPGS. In fact, specificity was higher for the identification of tumor invasion in the pPGS, where more adipose tissues, and the presence of cartilaginous structures, are providing more radiological signs (for example, the alteration of both arytenoid and thyroid cartilages with enlargement of the thyro-arytenoid distance). CT evaluation of PGS has been rarely addressed in the literature. In a recently published study, Jaipuria et al. [29] compared CT staging with histopathology in T3–T4 laryngeal cancers, reporting 85.7% sensitivity, 77.8% specificity and 82.6% accuracy for the assessment of anterior and posterior PGS. In the same study, thyroid cartilage early infiltration detection showed 86.7% sensitivity, 50% specificity and 65.2% accuracy.

Finally, CT detection of extralaryngeal spreads in our study showed a lower sensitivity in the anterior commissure and AVF (68.8% and 63.6%, respectively) as compared to the posterior compartment (100%). Specificity, instead, was higher in the anterior segments (91.5% in AC and 98.1% in AVF vs. 87.9% in PVF). This observation could not be confirmed by the literature data, as no other study, to the best of our knowledge, compared CT accuracy in anterior and posterior glottic tumors. Considering the average data, several studies reported similar or lower figures [18,19,20,21].

Over the last few years, attention has been focused on the capability of MRIs to detect cartilage tumor invasions in laryngeal compartments. In a recently published review, Cho et al. compared the accuracy of a CT with an MRI in the identification of cartilage infiltration: sensitivity resulted significatively higher in the MRI (90% vs. 66% for CT), while specificity was similar (81% for MRI vs. 88% for CT) [32]. The low specificity of MRIs described in older studies was later improved: considering a T2-weighted or postcontrast T1-weighted cartilage signal intensity greater than that of the adjacent tumor as a sign of inflammation, and a signal intensity similar to that of the adjacent tumor as a sign of neoplastic invasion, as proposed by Becker et al., resulted in a significant increase of specificity [33]. Recently, Ravanelli et al. [10] proposed a pattern of MRI interpretation that further improved specificity also in PGS: the identification of the T2 intermediate signal, high-diffusion weighted imaging (DWI) signal combined with the low apparent diffusion coefficient and the variable contrast enhancement strongly correlated with neoplastic invasion (sensitivity 100%, specificity 78%). Moreover, MRI diagnostic performances have considerably improved in recent years thanks to the use of dedicated surface coils which provide a higher spatial resolution in a short acquisition time [13]. Although an MRI provides a better discrimination of submucosal tissue changes, it has some disadvantages in terms of availability, costs, acquisition time and patient tolerance. Moreover, it does not permit a dynamic evaluation of the larynx, and degradation of the image quality related to motion artifacts is a common pitfall [9,11,13].

The survival analysis in our study showed that the pathological stage and histopathological evidence of pPGS tumor invasions, but not the CT study results, were significantly associated with DSS and DFS. This further supports the suboptimal accuracy of CT in tumor staging at this critical level and suggests the use of more accurate imaging tools when the involvement of the pPGS impacts on the surgical decision. A CT scan can be considered a useful tool to evaluate anterior cartilage infiltration, but, when a pPGS invasion is suspected (arytenoid hypomotility or fixation), an MRI offers a higher contrast resolution and is particularly helpful in distinguishing a tumor infiltration from a peritumoral inflammation and in depicting cartilage infiltration [9]. Considering our observation of a predominant over-staging of glottic cancer, it can be argued that the use of more specific parameters, as those provided by an MRI, can allow less aggressive surgical choices, increasing the number of OPHL with respect to TL and even of transoral laser microsurgery in selected cases.

## 4. Materials and Methods

This is a retrospective cohort study of locally advanced glottic SCC treated by TL or OPHL with no need to receive approval from ethics committee. All patients who received a curative laryngectomy for glottic SCC at the Otorhinolaryngology Department of the IRCCS Policlinico San Matteo Foundation, Pavia, Italy from January 2005 to August 2019 were recruited. Exclusion criteria were nonepithelial cancer, primary supraglottic or subglottic tumors and a lack of preoperative CT scans available for review.

**Figure 6:**
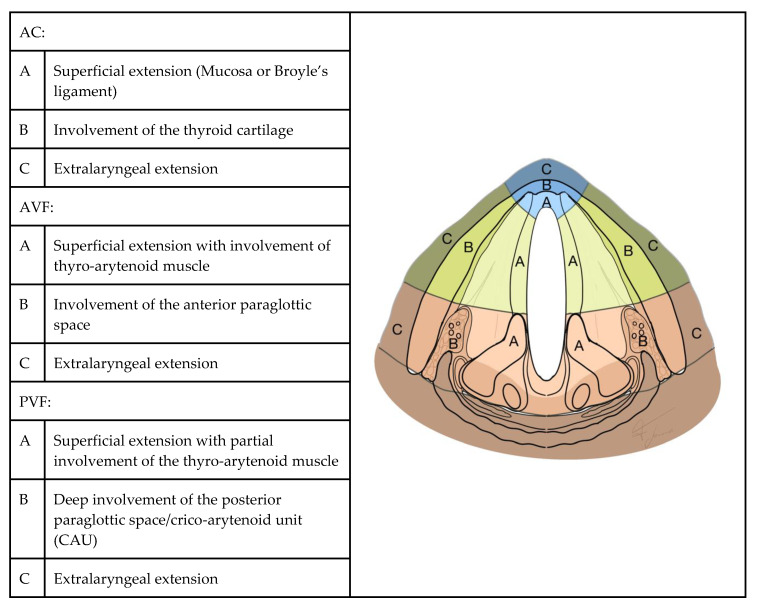
Glottic map: subsites and layers of depth. AC: anterior commissure, AVF: anterior vocal fold and PVF: posterior vocal fold.

Presurgical neck CT Digital Images and Communication in Medicine (DICOM)-format images of each patient were collected and reviewed by an experienced head and neck radiologist who was unaware of prior CT staging and of the final pathological diagnosis. All scans were acquired after intravenous administration of an iodine-based contrast medium; slice thickness ranged between 1 and 4 mm. Due to the wide enrollment time interval, and to the fact that CT scans had sometimes been performed in other hospitals, a largely heterogeneous series of scans was collected. Aware of these limitations, the radiologist was asked not only to confirm the previously assessed CT stage but also to identify the precise extension of the tumor in each direction of the glottic plane. To easily represent this information, we introduced a "radiological glottic map (rMAP)", an instrument that provides an immediate view of tumor spreading. In rMAP, we identified three different subsites of the glottic plane: anterior commissure (AC), anterior vocal fold region (AVF) and posterior vocal fold region (PVF). Each subsite was then divided into three layers of depth, as represented in Figure 6. Histopathological reports and slides were retrieved from the archives of the pathology unit and reviewed by 2 experienced head and neck pathologists who were unaware of prior CT staging. The same topographic criteria used for the definition of rMAPs (Figure 6) were applied to the evaluation of histopathological samples, in order to define a “pathological glottic map (pMAP)” to represent the extension and levels of invasion in individual topographic subsites. The two maps were then compared in every case, in order to identify over- and under-staging of extension and invasion at each subsite in rMAP as compared with the reference pMAP. Patient records were reviewed in order to determine oncological outcomes. We have identified as a radiological and histological criterion of AC lateral limit the anterior edge of tyro-arytenoid muscles in order to include in this region only three layers: superficial mucosa, Broyle’s ligament and thyroid cartilage. AVF, instead, is composed of 5 layers: mucosa, lamina propria (Reinke’s space and vocal ligament), tyro-arytenoid muscle, aPGS and thyroid cartilage. PVF anterior limit was defined according to a plane passing through the arytenoid vocal process, perpendicular to the ipsilateral thyroid lamina [2,3], and comprised mucosa, lamina propria, tyro-arytenoid muscle, pPGS, arytenoid and thyroid cartilage.

The diagnostic accuracy of the CT scan (rMAP) was determined considering the histopathological findings (pMAP) as the gold standard for the assessment of tumor extensions. In detail, specificity, sensitivity, positive and negative predictive value and accuracy of CT scans in assessing the presence of tumors and the level of tumor invasion (superficial tumor spread, whole thickness infiltration up to the thyroid cartilage and extralaryngeal extension) were measured at each glottic subsite (anterior commissure, anterior vocal fold and posterior vocal fold). Survival analysis was performed with the Cox univariate model, including the following parameters: clinical and pathological stage, presence of nodal metastases and invasion of the pPGS at CT and histopathological evaluation; a *p*-value < 0.05 was considered as significant. All statistical tests were performed using the MedCalc application (www.medcalc.be). 

## 5. Conclusions

Accurate preoperative staging of laryngeal cancer is mandatory for both prognostic and surgical planning purposes. In particular, a pathological evidence of neoplastic pPGS involvement has a negative prognostic correlation, which is not considered in the current TNM staging system. CT low-contrast resolution with soft tissues limits the evaluation of deep submucosal compartments, inducing an overall over-estimation of tumor invasion, especially in areas that are critical for surgical decision, such as the pPGS, as confirmed by the comparison with histopathological staging. Since the introduction of new technologies and diagnostic algorithms has improved the diagnostic performance of surface-coil MRI with respect to CT in the evaluation of the larynx, its use should be advised when the involvement of the PGS is suspected.

## Figures and Tables

**Table 1:** Radiological and histopathological assessments of tumor extensions.

**cT**	**N (%)**	**pT**	**N (%)**
cT2	4 (6.3%)	pT2	19 (29.7%)
cT3	39 (60.9%)	pT3	28 (43.8%)
cT4a	21 (25.0%)	pT4a	16 (25.0%)
**Preoperative radiological staging**	**N (%)**	**Histopathological staging**	**N (%)**
cII	4 (6.3%)	pII	18 (27.7%)
cIII	37 (57.8%)	pIII	25 (38.5%)
cIVA	23 (35.9%)	pIVA	1 (1.5%)
cIVB	0 (0.0%)	pIVB	4 (6.2%)
**rMAP**	**N (%)**	**pMAP**	**N (%)**
**AC**	43 (62.3%)	**AC**	50 (78.1%)
A	15 (21.7%)	A	21 (32.8%)
B	13 (18.8%)	B	13 (20.3%)
C	15 (21.7%)	C	16 (25.0%)
**AVF**	63 (91.3%)	**AVF**	63 (98.4%)
A	6 (8.7%)	A	20 (31.3%)
B	49 (71.0%)	B	32 (50.0%)
C	8 (11.6%)	C	11 (17.2%)
**PVF**	32 (46.4%)	**PVF**	34 (53.1%)
A	3 (4.4%)	A	6 (9.4%)
B	16 (23.2%)	B	22 (34.4%)
C	13 (18.8%)	C	6 (5.1%)

AC: anterior commissure, AVF: anterior vocal fold, PVF: posterior vocal fold, AJCC: American Joint Committee on Cancer, rMAP: radiological glottic map and pMAP: histopathological glottic map.
